# An Account of the Cases of Dislocation of the Femur at the Hip Joint Treated by Manipulation Alone, after the Plan Proposed by W. W. Reed, M. D., of Rochester, Which Have Occurred in the New York Hospital during the past Two Years

**Published:** 1855-05

**Authors:** Thos. M. Markoe

**Affiliations:** The Attending Surgeons


*An Account of the Cases af Dislocation of the Femur at the Hip Joint treated by Manipulation alone, after the plan proposed* by W. W. Reed, M. D., of Rochester, which have occurred in the New York Hospital during the past two years. By Thos. M. Markoe, M. D., one of the Attending Surgeons.—Dr. Markoe relates 14 cases of luxation of the hip joint, all of them treated on the principle of Dr. Reed's plan, that dislocation of the femur on the dorsum ilii can be remedied "by flexing the leg on the thigh, carrying the thigh over the sound one, upwards over the pelvis, as high as the umbilicus, and then by abducting and rotating it." After narrating thirteen cases, the writer says: "The cases which are related embrace every instance of dislocation of the femur which has presented itself in the Hospital during the last two years, and a reduction was always effected without the aid of pullies, excepting in the one case of Dr. Halsted, No. 7, and here a modification of the manipulation which had previously proved successful was not tried. In Dr. Post's case, No. 4, the reduction was finally effected from the foramen ovale by the usual mode, though without the pullies, but it was brought by the manipulation employed to this point from the ischiatic notch, from which it surely would have required the pullies to displace it on the old plan." "Though we have failed then, in reducing, by the new plan, two cases out of the thirteen, yet I think that the record shows good reason for believing that all might have been so reduced, if we had known at the time as much as subsequent cases have taught us." A rocking motion of the limb upon the pelvis, moving the leg backward and forward, frequently has the effect, when the head is near the acetabulum, of making it enter the socket. In seven of the cases the luxation was upon the dorsum ilii, three were into the ischiatic notch, and three were into the foramen ovale. Dr. Markoe states, that when the head of the bone is brought down towards the lower portion of the acetabulum, where its margin is least prominent, the mode of procedure had to be varied, sometimes, from that of Dr. Reed's. "He recommends, when the head is brought by abduction close to the lower edge of the acetabulum, that, by the rocking movement already described, it be caused to slip in. This is well, and will probably answer in many cases, but it failed so completely from the first, that we were led to add the bringing down of the thigh to the straight position in a state of abduction, still keeping up the rocking motion, and it has been uniformly in the act of thus bringing down the limb, that the reduction has been accomplished." The continuance of the movement, without force, is all that is required. After writing out the thirteen cases above mentioned, Dr. Markoe states that a case of luxation on the dorsum ilii came into the hospital. After trying Dr. Reed's plan several times without success, the usual method recommended by Sir Astley Cooper was adopted. A fracture of the cervix was the consequence.

The principle of Dr. Reed's plan in its application to the different forms of dislocation, presents some variations. It has its best illustrations in dislocations on the dorsum and into the ischiatic notch, their mechanism being for the purpose identical.

Dr. Markoe's paper is a most excellent one, and should be studied by every surgeon in the country.

